# Host–Microbiome Immune Interaction Networks: A Comparative Evolutionary Perspective Across Worms, Mice, and Humans

**DOI:** 10.3390/pathogens15070733

**Published:** 2026-07-13

**Authors:** Xuanheng Tai, Yiying Zhang, Huijie Yang, Wei Zou

**Affiliations:** Yunnan Provincial Key Laboratory of Public Health and Biosafety, School of Public Health, Kunming Medical University, Kunming 650500, China; ttll22007@163.com

**Keywords:** holobiont, microbiome–immune interactions, evolutionary mismatch, immune homeostasis, surveillance immunity, and precision medicine

## Abstract

The immunological paradigm is undergoing profound shifts. Classic “self-nonself” recognition remains foundational, but microbiome research has expanded immunological models toward homeostasis, tolerance, and host–microbe co-regulation. Within this framework, gut microbiota act as the core drivers of the development and calibration of the host immune system. This review examines host–microbiome immune interactions across species from a macroevolutionary perspective. We use *C. elegans*, mouse models, and humans as comparative systems representing different levels of immune complexity and translational relevance. These include *Caenorhabditis elegans* (primitive innate immunity via cytosolic surveillance), mouse models (microbiota-driven shaping of adaptive immunity and homeostatic trade-offs), and the human system (modern lifestyle-induced “evolutionary mismatch” and inflammatory diseases). The immune system has evolved significantly over time. It has transitioned from independent cellular integrity monitoring to a complex network that is heavily reliant on external microbial cues. This potential integration of developmental signals from commensal microbes enhances environmental adaptability but may also increase host susceptibility to inflammatory disorders under modern ecological shifts. Deconstructing this cross-species interaction blueprint offers significant theoretical value. Furthermore, it provides the guiding logic for developing next-generation precision microbiome therapies that target the holobiont, such as engineered microbial consortia and personalized postbiotic interventions.

## 1. Introduction

For a long time, the logical starting point of immunology has been anchored in Burnet’s “self-nonself” recognition model. This model views the clearance of foreign organisms as the primary function of the immune system [1]. However, the explosive growth of gut microbiome research has fundamentally challenged this traditional dogma. Modern evidence shows that the vast microbial community in the human gut is not simply a target of defense. Instead, it is an indispensable collaborator of the immune system [2]. The current theoretical framework has shifted toward a “homeostasis maintenance” model. This emphasizes that the immune system must strike a delicate, dynamic balance between “resistance” against pathogens and “tolerance” towards commensal bacteria [3]. A breakdown of this balance is a major contributing factor in the pathogenesis inflammatory bowel disease (IBD) and various autoimmune disorders. Understanding this shift requires introducing the revolutionary concept of the “holobiont.” This theory proposes that the multicellular host and its symbiotic microbiota form a unified evolutionary unit. While the holobiont concept serves as a useful conceptual framework for host–microbe co-regulation, it remains debated regarding the heritability of the microbiota, the stability of specific microbial partners, and whether selection operates primarily at the level of the individual host or the symbiotic collective [4]. From this perspective, the host immune genome does not exist in isolation but is deeply intertwined with the host microbiome. Gut microbes continuously provide ligand signals (such as microbe-associated molecular patterns (MAMPs)) and metabolic intermediates (such as SCFAs). Through this, they directly participate in the development of immune organs and functional calibration of immune cells [5]. This strategy of “co-evolutionary integration of developmental signals” significantly enhances the adaptability and robustness of the holobiont under complex environmental pressures [6].

To systematically deconstruct the conserved rules of this symbiotic architecture, this study compares three strategically significant nodes. First, *Caenorhabditis elegans* (*C. elegans*) serves as a foundational model. It demonstrates how a host lacking specialized lymphocytes relies solely on a single-layer intestinal epithelium to perform precise “physiological surveillance,” triggering defenses by sensing core metabolic damage [7]. Second, *Mus musculus* represents the pinnacle of integrating innate and adaptive immunity in mammals. It provides a core platform for studying how microbes (such as SFB) precisely educate naive T cells and induce Th17 differentiation [8]. Finally, *Homo sapiens* studies have revealed the phenomenon of “evolutionary mismatch” caused by drastic modern lifestyle changes. This collapse of the ancient symbiotic pact has triggered a global epidemic of inflammatory diseases. By comparing these strategic nodes, we aim to outline a comprehensive blueprint of host–microbe defense architectures across different biological scales [9].

### Search Strategy and Selection Criteria

This article is a narrative review Literature for this review was identified through searches of PubMed, Web of Science, and Google Scholar for articles published up to January 2026. Search terms included combinations of “surveillance immunity”, “*C. elegans*”, “segmented filamentous bacteria (SFB)”, “Th17/Treg balance”, “*Bacteroides fragilis*”, “inflammatory bowel disease (IBD)”, “multiple sclerosis (MS)”, “fecal microbiota transplantation (FMT)”, and “postbiotics”. Articles were prioritized based on their mechanistic rigor, reliance on peer-reviewed primary data, and translational relevance. We focused on well-established models (e.g., SFB and *B. fragilis*) to illustrate general biological principles of host–microbe calibration.

## 2. The Primitive Blueprint of Innate Immunity: Defense Networks of *C. elegans*

### 2.1. Pathogen-Induced Damage Surveillance and Epithelial Defense

*C. elegans* provides a valuable model for exploring the evolutionary starting point of defense architectures. Lacking specialized free-living immune cells, its defense line is concentrated in the “epithelium-intrinsic defense system” [10]. Unlike mechanisms that rely on PRRs for direct microbial recognition, the *C. elegans* has evolved a unique “surveillance immunity” strategy. It indirectly senses pathogen-induced damage by monitoring the integrity of core physiological processes, such as the unfolded protein response (UPR) and endoplasmic reticulum (ER) stress [11,12].

Key triggers include translational inhibition (sensed by the transcription factor ZIP-2), membrane integrity/lysosomal stress (monitored by HLH-30/TFEB), and endoplasmic reticulum (ER) stress [11,12]. Within this network, the conserved triple kinase cascade (NSY-1–SEK-1–PMK-1) acts as the core signal amplifier [13]. Phosphorylated PMK-1 activates the bZIP transcription factor ATF-7, releasing its repression of antimicrobial peptide (AMP) genes to initiate defense [14]. Notably, while the *C. elegans* relies heavily on “cytoplasmic surveillance,” the defense architecture did not directly leap to mammals. As a key evolutionary bridge, arthropods, such as *Drosophila*, later developed the classic Toll/IMD pathways to recognize microbial structural motifs. These pathways provided the necessary foundation for the complex PRR networks found in higher mammals [15].

### 2.2. Commensal Metabolic Signaling and Epigenetic Homeostasis

High-intensity AMP expression triggers significant metabolic trade-offs and tissue stress. This may even impair host homeostasis. To prevent immune overload, *C. elegans* utilizes precise negative feedback mechanisms, for instance, the MAPK phosphatase VHP-1 dephosphorylates PMK-1 to terminate the response [16]. Additionally, XBP-1, a key factor in the UPR pathway, coordinates with PMK-1 to maintain proteostasis and balance immunity with cell survival [17].

Microbes are not merely “monitored threats” but vital communication partners. Research has shown that metabolites, such as nitric oxide (NO), from commensal bacteria can diffuse across kingdoms. These signals target and activate host transcription factors, HSF-1 and DAF-16, enhancing stress resistance and lifespan [18]. This metabolic signal integration reflects the deep coupling between immunity and metabolism. Furthermore, recent studies have revealed that this inter-kingdom communication, particularly through small RNAs or metabolic signals, establishes “transgenerational immune memory” through specific histone modifications, such as H3K4me3 [19]. This primitive epigenetic calibration in *C. elegans* foreshadows the complex logic in mammals, where short-chain fatty acids (SCFAs) maintain regulatory T (Treg) cell homeostasis by regulating histone deacetylases (HDACs) [20].

### 2.3. Neuroimmune Integration Systems and Behavioral Defense

**Figure 1:**
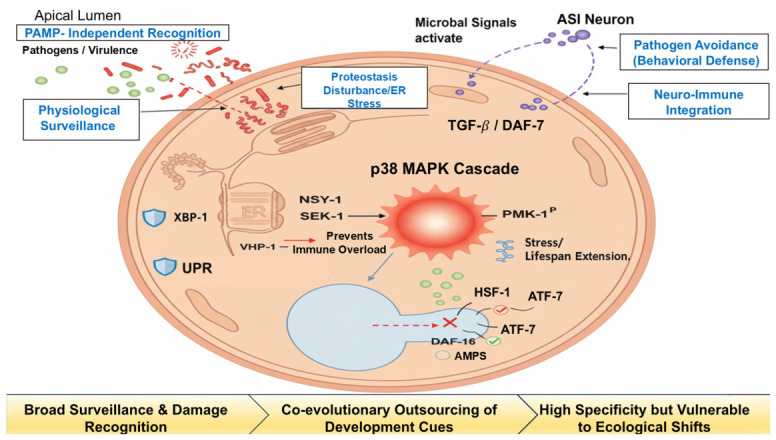
Integrated surveillance immunity and neuro-endocrine defense network in *C. elegans*. Abbreviations: AMPs, antimicrobial peptides; ASI, amphid single-ciliated neuron; ATF-7, activating transcription factor 7; DAF, Dauer formation-related; ER, endoplasmic reticulum; HSF, heat shock factor; MAPK, mitogen-activated protein kinase; NSY-1, neuronal symmetric 1; PAMP, pathogen-associated molecular pattern; PMK-1, p38 MAPK family; SEK-1, SAPK/ERK kinase 1; TGF-β, transforming growth factor-beta; UPR, unfolded protein response; VHP-1, VH1-like phosphatase 1; XBP-1, X-box binding protein 1. Figures were created with Figdraw 2.0.The figure illustrates the multi-layered defense architecture of *C. elegans*, transitioning from local epithelial surveillance to systemic neuro-immune coordination. Pathogen-induced physiological disruptions (e.g., ER stress and translational inhibition) are sensed via “surveillance immunity,” bypassing traditional PAMP recognition. The p38 MAPK cascade (NSY-1–SEK-1–PMK-1) serves as the central defense hub, where phosphorylated PMK-1 de-represses ATF-7 to drive antimicrobial peptide (AMP) expression. Homeostasis is maintained by the VHP-1 phosphatase (negative feedback) and the UPR/XBP-1 pathway (proteostasis). Simultaneously, commensal microbial signals activate DAF-16 and HSF-1 to enhance stress resilience. Systemic coordination is achieved through the ASI neuron, which secretes the TGF-β-like ligand DAF-7 to remotely regulate intestinal immunity and trigger learned pathogen avoidance behavior. The bottom chevrons trace the evolutionary trajectory from broad-spectrum damage recognition to complex host-microbe metabolic integration. Color coding: Red red arrows and symbols indicate pathogen-driven activation or damage; blue shields represent cytoprotective/homeostatic mechanisms; purple dashed lines denote neuro-endocrine regulatory axes; and green elements highlight beneficial commensal-mediated signaling.

In addition to local biochemical defenses in the intestinal epithelium, *C. elegans* exhibits highly developed cross-organ integration. This is particularly evident in neuro-immune regulation. Specific environmental microbial signals are sensed by ASI neurons in the head and converted into endocrine signals, inducing the secretion of the TGF-β-like ligand DAF-7 from ASI neurons [21]. This neuro-endocrine axis remotely regulates intestinal immunity and drives “learned pathogen avoidance” behavior. By allowing the organism to “recognize” and bypass harmful bacteria based on prior exposure, this forms a vital part of behavioral immunity [22]. Neural signals also influence intestinal AMP expression and stress response levels by modulating p38 MAPK pathway activity. This dual linkage of behavioral defense and molecular immunity represents a primitive “gut–brain axis” [23]. It couples external sensory-driven behavioral defense with internal physiological immunity into a single organism-wide network. This reflects the integrated strategy used by early animals for efficient adaptation in complex environments (Figure 1).

### 2.4. Limitations of Extrapolating C. elegans to Mammals

While *C. elegans* provides an elegant model for cell-intrinsic surveillance, direct extrapolation to mammalian systems is constrained. Lineages leading to nematodes lacks mobile phagocytes, specialized mucosal lymphoid organs, and adaptive immune elements (e.g., T/B lymphocytes and immunoglobulins). Mammalian mucosal immunity is significantly more complex, managing a highly diverse commensal microbiota through specialized cellular networks.

The figure illustrates the multi-layered defense architecture of *C. elegans*, transitioning from local epithelial surveillance to systemic neuro-immune coordination. Pathogen-induced physiological disruptions (e.g., ER stress and translational inhibition) are sensed via “surveillance immunity,” bypassing traditional PAMP recognition. The p38 MAPK cascade (NSY-1–SEK-1–PMK-1) serves as the central defense hub, where phosphorylated PMK-1 de-represses ATF-7 to drive antimicrobial peptide (AMP) expression. Homeostasis is maintained by the VHP-1 phosphatase (negative feedback) and the UPR/XBP-1 pathway (proteostasis). Simultaneously, commensal microbial signals activate DAF-16 and HSF-1 to enhance stress resilience. Systemic coordination is achieved through the ASI neuron, which secretes the TGF-β-like ligand DAF-7 to remotely regulate intestinal immunity and trigger learned pathogen avoidance behavior. The bottom chevrons trace the evolutionary trajectory from broad-spectrum damage recognition to complex host–microbe metabolic integration. Color coding: red arrows and symbols indicate pathogen-driven activation or damage; blue shields represent cytoprotective/homeostatic mechanisms; purple dashed lines denote neuro-endocrine regulatory axes; and green elements highlight beneficial commensal-mediated signaling.

## 3. Microbiota-Dependent Immune Shaping in Mouse Models

### 3.1. Anatomical Development of Gut Immune Tissues and Microecological Instructional Signals

Gut-associated lymphoid tissue (GALT) forms the core anatomical basis for mucosal immune surveillance. However, its formation and functional maturation are not entirely determined by the host’s intrinsic programs. Instead, they rely heavily on continuous shaping by postnatal microbial signals. In other words, the maturation of gut immune structures is fundamentally a dynamic process driven synergistically by “developmental programs” and “environmental inputs” [24]. Peyer’s patches (PPs) serve as an example. Their primordia are established during embryonic development. However, the formation of fully immunocompetent germinal centers, along with B cell activation and affinity maturation, clearly depends on postnatal gut microbiota colonization and antigen stimulation [25].

In contrast, the development of isolated lymphoid follicles (ILFs) is even more environmentally dependent. In germ-free (GF) mice, ILFs typically remain in an immature state of lymphocyte aggregation [26]. Only when the host senses microbe-derived signals like peptidoglycan through pattern recognition systems, and subsequently activates the lymphotoxin (LT) pathway, is the remodeling and functionalization of follicular structures triggered.

This process suggests that gut immune tissues are not static structures. They undergo “plastic regulation” in response to changes in microbial load and composition. This achieves a dynamic balance between the physical barrier and immune surveillance. At the effector level, this structural plasticity is further reflected in the generation modes of secretory IgA (sIgA) [27,28]. High-affinity IgA is primarily produced in PPs through a T cell-dependent (TD) pathway to precisely clear specific pathogens. In contrast, the lamina propria mainly relies on a T cell-independent (TI) pathway. Driven by BAFF/APRIL signaling, this pathway produces broad-spectrum IgA to restrict the over-expansion of commensal bacteria through multivalent binding [29,30]. Therefore, from lymphoid structural development to antibody production methods, the gut immune system demonstrates a deep reliance on microbial signals. This coordinated regulation of structure and function builds the first line of defense for maintaining mucosal homeostasis.

### 3.2. Directed Induction and Polarization of Helper T Cells by Specific Commensal Microbiota

At the level of adaptive immunity, gut microbes provide more than just antigen stimulation. They can selectively shape the direction of T cell differentiation. Segmented filamentous bacteria (SFB) serve as a classic model for driving Th17 cell differentiation [31]. SFB tightly adhere to the surface of the ileal epithelium. This induces epithelial cells to secrete serum amyloid A (SAA). SAA activates lamina propria dendritic cells (DCs) to produce IL-6 and IL-23, which propels naive CD4^+^ T cells to differentiate into the Th17 lineage [32,33]. This “directed polarization” mediated by specific commensal bacteria significantly enhances host defense against intestinal pathogens (such as *Citrobacter rodentium*). However, the Th17 response is not unilaterally beneficial. The IL-17A/F and IL-22 secreted by Th17 cells play critical roles in strengthening the epithelial barrier and inducing antimicrobial peptide expression. While gut-resident Th17 cells primarily coordinate homeostatic defense and barrier integrity, ectopic or genetically permissive Th17 activation has been associated with autoimmune phenotypes in selected mouse models [34,35]. For instance, in individuals with specific genetic predispositions, the pathological potential of the SFB-induced Th17 pool is highly context-dependent, relying on the host genetic background, specific disease models, and the presence of co-occurring inflammatory cues. In genetically susceptible mice, these cells may contribute to experimental autoimmune encephalomyelitis (EAE) or arthritis, whereas in wild-type mice under homeostatic conditions, they primarily support barrier integrity without causing systemic pathology.

Therefore, SFB-driven Th17 polarization highlights the context-dependent nature of microbe–immune interactions. It demonstrates a delicate boundary between local protective immunity and systemic immunopathology, in which the host balances efficient mucosal defense against the risk of breaking self-tolerance in extra-intestinal tissues [36].

### 3.3. Epigenetic Calibration of Immunosuppressive Signals and Metabolites

Parallel to pro-inflammatory responses, gut commensal bacteria play an indispensable role in establishing immune tolerance. They act as the “braking system” for maintaining immune homeostasis. *Bacteroides fragilis* is a representative species in this regard. It secretes polysaccharide A (PSA), which induces IL-10 production via the TLR2 signaling pathway. This promotes the differentiation of regulatory T cells (Tregs), thereby suppressing excessive inflammatory responses and maintaining mucosal immune balance [37,38].

In addition to direct receptor–ligand signaling, microbial metabolites exert a deeper influence on immune regulation. Short-chain fatty acids (SCFAs), particularly butyrate, participate in anti-inflammatory signaling by activating G protein-coupled receptors (such as GPR43). Furthermore, butyrate inhibits histone deacetylase (HDAC) and directly affects chromatin structure. By increasing the acetylation level of the *Foxp3* gene promoter region, butyrate stably promotes the differentiation and maintenance of peripheral Treg cells at the epigenetic level [39,40].

**Figure 2:**
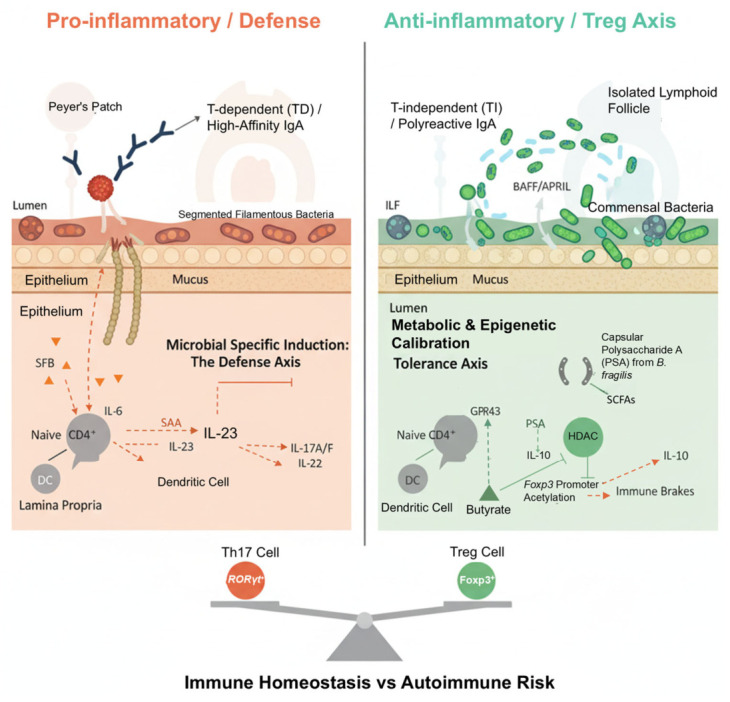
Orchestration of the mammalian adaptive immune balance by gut microbiota and metabolites. Abbreviations: APRIL, a proliferation-inducing ligand; BAFF, B-cell activating factor; DC, dendritic cell; Foxp3, forkhead box P3; GPR43, G protein-coupled receptor 43; HDAC, histone deacetylase; IgA, immunoglobulin A; IL, interleukin; ILF, isolated lymphoid follicle; PSA, polysaccharide A; RORγt, retinoic acid receptor-related orphan nuclear receptor gamma t; SAA, serum amyloid A; SCFA, short-chain fatty acid; SFB, segmented filamentous bacteria; TD, T-dependent; Th17, T helper 17; TI, T-independent; Treg, regulatory T. Figures were created with Figdraw. The figure depicts the divergent pathways through which commensal microbes shape the T helper 17 (Th17) and regulatory T (Treg) cell landscape landscapes in the intestinal lamina propria. On the pro-inflammatory/defense axis (left), Segmented Filamentous Bacteria segmented filamentous bacteria (SFB) adhere to the epithelium, inducing the secretion of Serum Amyloid serum amyloid A (SAA). This triggers the the IL-6/IL-23 signaling cascade in dendritic cells (DCs), driving the polarization of naive CD4+ T cells into into RORγt+ Th17 cells and promoting high-affinity, T-dependent (TD) IgA production in Peyer’s patches. On the anti-inflammatory/tolerance axis (right), commensals, commensals like such as *B. fragilis*, and their metabolites (e.g., butyrate) promote immune homeostasis. PSA and SCFAs signal through GPR43 and TLR2 to induce induce IL-10-secreting secreting Foxp3+ Treg cells. Mechanistically, butyrate serves as a a histone deacetylase (HDAC) inhibitor, increasing acetylation of the *Foxp3* the *Foxp3* promoter to stabilize the Treg phenotype. The central balance illustrates the homeostatic equilibrium between these two hubs; a shift toward excessive Th17 activation increases the risk of autoimmunity. Color Coding: Orange elements represent the pro-inflammatory defense axis triggered by specific pathobionts (e.g., SFB). Green elements highlight the anti-inflammatory tolerance axis driven by commensal metabolites and epigenetic recalibration. Solid lines indicate direct cellular differentiation pathways, while whereas dashed lines trace cytokine signaling and metabolic diffusion.

This process suggests that microbes do not merely regulate immune responses through “signaling molecules.” They also remodel host gene expression programs through “metabolites,” converting the metabolic state of the gut into a long-term, stable immune phenotype. Consequently, host–microbe interactions are no longer confined to short-term signal responses. They extend to the level of epigenetic regulation, forming a crucial foundation for maintaining immune homeostasis (Figure 2) [41].

The figure depicts the divergent pathways through which commensal microbes shape the T helper 17 (Th17) and regulatory T (Treg) cell landscapes in the intestinal lamina propria. On the pro-inflammatory/defense axis (left), segmented filamentous bacteria (SFB) adhere to the epithelium, inducing the secretion of serum amyloid A (SAA). This triggers the IL-6/IL-23 signaling cascade in dendritic cells (DCs), driving the polarization of naive CD4+ T cells into RORγt+ Th17 cells and promoting high-affinity, T-dependent (TD) IgA production in Peyer’s patches. On the anti-inflammatory/tolerance axis (right), commensals, such as *B. fragilis*, and their metabolites (e.g., butyrate) promote immune homeostasis. PSA and SCFAs signal through GPR43 and TLR2 to induce IL-10-secreting Foxp3+ Treg cells. Mechanistically, butyrate acts as a histone deacetylase (HDAC) inhibitor, increasing acetylation of the Foxp3 promoter to stabilize the Treg phenotype. The central balance illustrates the homeostatic equilibrium between these two hubs; a shift toward excessive Th17 activation increases the risk of autoimmunity. Color Coding: Orange elements represent the pro-inflammatory defense axis triggered by specific pathobionts (e.g., SFB). Green elements highlight the anti-inflammatory tolerance axis driven by commensal metabolites and epigenetic recalibration. Solid lines indicate direct cellular differentiation pathways, whereas dashed lines trace cytokine signaling and metabolic diffusion.

## 4. Clinical Complexity of the Modern Human System and Barriers in Translational Medicine

### 4.1. The Mucosal Developmental Window and Functional Redundancy of the Human Immune Axis

Segmented filamentous bacteria (SFB) exhibit potent immune-inducing activity in mouse models. However, their colonization rate in the human gut is extremely low and is often limited to infancy [42,43]. This interspecies difference suggests a key evolutionary adaptation. The human immune system has recruited other commensal taxa with “functional redundancy” [44]. Current research in gnotobiotic animal models suggests that specific human-derived *Bacteroides* members have the potential to trigger SFB-like pro-Th17 differentiation effects, presumably by adhering to intestinal epithelial cells [45]. They help establish the “basal activation threshold” of mucosal immunity.

During this process, maternal breast milk-derived secretory IgA (sIgA) plays a critical rate-limiting regulatory role. sIgA mediates spatial steric hindrance against potential pathogens. It also neutralizes potent immune-inducing bacteria. This precisely controls the maturation rhythm of the infant mucosal immune system, thereby avoiding premature immune responses [46]. This delicate regulation relies on a specific developmental window. It profoundly illustrates the transgenerational role of maternal signals in human “microbial immune education.” However, early microbial perturbations may carry long-term risks of immune deviation [47].

### 4.2. Host Genetic Heterogeneity and Mechanisms of Dysbiosis in Inflammatory Bowel Disease

The pathological progression of inflammatory bowel disease (IBD) is fundamentally the collapse of the immune tolerance pact within the holobiont architecture. Human IBD pathogenesis represents a complex multifactorial process, in which genetic susceptibility (such as *NOD2* and *ATG16L1* mutations) and environmental triggers integrate to alter epithelial barrier integrity and mucosal immune homeostasis [48]. Clinical genomics has further revealed how these specific host susceptibility gene mutations impair intestinal epithelial perception of microbial signals and intracellular antibacterial degradation functions [49].

Against this backdrop of genetic susceptibility, modern lifestyle ecological pressures (such as exposure to Western diets and broad-spectrum antibiotics) act as important contributors that are associated with the depletion of gut butyrate-producing bacteria. At the molecular level, this depletion impairs key epigenetic nodes in intestinal epithelial cells (IECs). Butyrate acts as a class I histone deacetylase (HDAC) inhibitor; its loss reduces histone H3 acetylation near the promoters of barrier-promoting genes (such as those encoding claudin-1 and mucin-2). Concurrently, inositol phosphate metabolism acts as a metabolic sensor linked to HDAC activity in IECs. Dysregulation of the inositol–HDAC pathway impairs epithelial cell differentiation and weakens mucosal inflammatory tone, potentially compromising barrier integrity with or without overt shifts in microbial composition [50,51].

### 4.3. Cross-Kingdom Microbiome Reconstruction and Super-Donor Characteristics in Fecal Microbiota Transplantation

Fecal microbiota transplantation (FMT) represents a therapeutic strategy with distinct tiers of clinical validation. While it stands as an established clinical intervention with robust evidence for recurrent *Clostridioides difficile* infection (CDI), its application for reshaping the holobiont microecosystem in inflammatory bowel disease (IBD) remains an active area of clinical investigation with variable outcomes [52]. The “super-donor” phenomenon, systematically described by Haifer et al., reveals significant individual differences in transplantation success rates [52], although the super-donor effect is highly dependent on the recipient’s baseline ecological niche and immunological compatibility. As this concept has deepened, modern microbial ecology has moved beyond a purely bacterio-centric perspective. It now emphasizes the core contribution of the “trans-kingdom microbiome” [53].

A super-donor can significantly improve a recipient’s “ecosystem restoration” success rate. This relies not only on the enrichment of efficient SCFA-producing bacteria but also on the metabolite output capacity and immunomodulatory potential. More importantly, it heavily depends on the characteristics of specific gut viromes (especially the active phageome) and mycobiomes present in feces [54]. Specific bacteriophage networks can target and lyse pro-inflammatory pathogens or mediate horizontal gene transfer. This profoundly reshapes the recipient’s gut niche while promoting the colonization of beneficial bacteria. These findings provide a cutting-edge evaluation dimension for standardizing the definition of a “premium donor”, while recognizing that FMT failures are often attributed to recipient-specific engraftment resistance, diet-driven incompatibility, or host immune barriers [55].

While FMT has achieved regulatory and clinical maturity for recurrent *Clostridioides difficile* infection (CDI), high-quality randomized trial evidence for inflammatory bowel disease (IBD) and other immune-mediated indications remains mixed and context-dependent.

### 4.4. Gut–Brain Axis Dysbiosis and Central Nervous System Autoimmune Pathology

Crosstalk between the human gut and central nervous system (CNS) does more than set physiological thresholds. It actively drives the progression of systemic autoimmune diseases [56]. Multiple sclerosis (MS) is an example of an autoimmune disease. Microbiota-driven molecular mimicry has been proposed as a possible mechanism contributing to central nervous system (CNS) autoimmunity. While structural homology between specific gut commensal epitopes and host myelin basic protein (MBP) can cause cross-reactive T-cell activation in animal models [57], MS pathogenesis in humans is multifactorial—integrating genetic susceptibility, environmental triggers, viral factors (such as Epstein–Barr virus), and complex CNS-specific regulatory pathways [58] (Table 1).

Simultaneously, gut-derived inflammatory cytokines and molecules, such as LPS, can impact BBB integrity via systemic circulation. This further amplifies the central immune activation. This phenomenon poses a critical danger. When faced with an “evolutionary mismatch,” conserved signals originally meant to sense the gut microenvironment can transform into an evolutionary trap, leading to systemic self-destruction [59].

**Figure 3:**
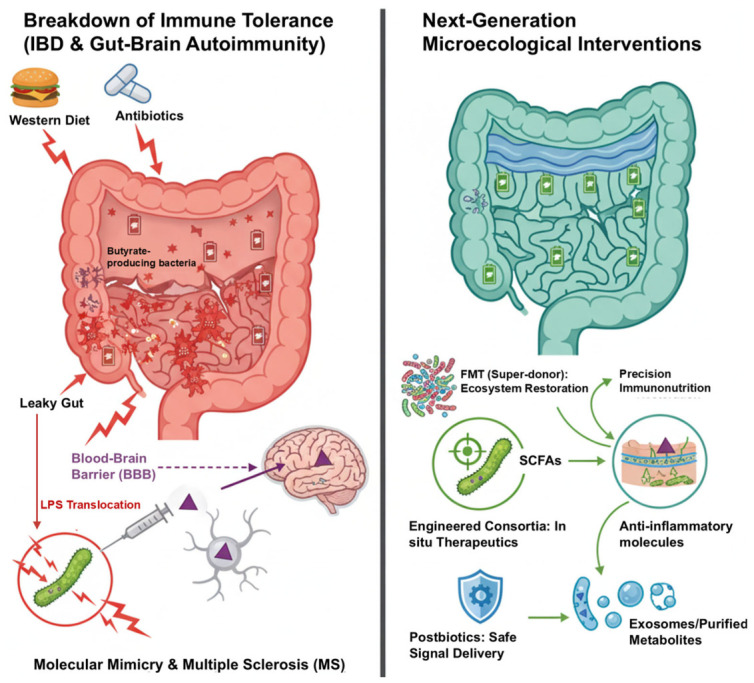
From evolutionary mismatch to precision medicine: pathological mechanisms and next-generation interventions. Abbreviations: BBB, blood–brain barrier; FMT, fecal microbiota transplantation; IBD, inflammatory bowel disease; LPS, lipopolysaccharide; MS, multiple sclerosis; SCFAs, short-chain fatty acids. Figures were created with Figdraw. The left panel illustrates the systemic decompensation triggered by “evolutionary mismatches.” Modern environmental stressors, specifically Westernized diets and Future Perspectives on Precision Microbiome Therapy antibiotic overuse, act as disruptive triggers (lightning bolts) that deplete the ancestral butyrate-producing bacteria. This metabolic exhaustion (indicated by low battery icons) compromises epithelial energy supply and mucus integrity, resulting in a “leaky gut” state. Subsequent systemic translocation of lipopolysaccharide (LPS) and pro-inflammatory mediators facilitates the penetration of the blood-brain barrier (BBB). In genetically susceptible hosts, this neuro-immune crosstalk can drive autoimmune pathologies, such as multiple sclerosis, which is often exacerbated by molecular mimicry between microbial antigens and neural proteins. The right panel depicts next-generation microecological strategies designed to repair ruptured symbiotic pacts. Ecosystem-level restoration is achieved through fecal microbiota transplantation (FMT) using “super-donor” profiles, complemented by precision immunonutrition to replenish metabolic niches. At the molecular level, engineered microbial consortia serve as in situ factories for the targeted release of short-chain fatty acids (SCFAs) and other anti-inflammatory effector molecules. Furthermore, postbiotics (including exosomes and purified metabolites) offer a cell-free, pharmacokinetically controllable pathway for the direct delivery of safe immunomodulatory signals to the host mucosal and systemic interfaces. Visual Logic: Red arrows and icons denote the pathobiont-driven inflammatory cascade; green elements symbolize the restoration of metabolic homeostasis and immune tolerance.

Furthermore, the neurological consequences of barrier disruption extend beyond demyelinating diseases like MS. Recent studies suggest that increased intestinal permeability allows systemic translocation of pro-inflammatory bacterial products, which can perturb vagus nerve–mediated gut–brain communication. This chronic inflammatory signaling along the gut–brain axis has been implicated in altering microglial activity and accelerating protein aggregation in brain regions associated with the early stages of neurodegenerative disorders, including Alzheimer’s and Parkinson’s diseases (Figure 3).

### 4.5. Next-Generation Microphysiological Research Models for Breaking Species Translation Bottlenecks

The ultimate goal of holobiont medicine is to achieve clinical translation. However, the immense genetic heterogeneity of the human system, coupled with the inability to perform arbitrary gene knockouts, forms a core barrier to translating findings from mice to the clinic [60]. To solve this problem, research paradigms are accelerating toward next-generation human-specific in vitro and in vivo models.

By colonizing germ-free mice with specific human patient microbiota, researchers create “humanized gnotobiotic mice.” This allows the accurate simulation of dysbiotic phenotypes that reflect human heterogeneity. Concurrently, scientists are utilizing patient-derived intestinal organoids and “gut-on-a-chip” microphysiological systems. These tools enable high-throughput analysis of the interaction dynamics among specific commensal strains, metabolites (such as SCFAs), and human immune cells. Crucially, this is achieved within a three-dimensional space that highly replicates the human mucosal barrier, fluid shear stress, and immune microenvironment [61,62]. However, these advanced in vitro models carry inherent limitations. They currently lack the complete systemic physiological complexity of a living host, have simplified immune cell repertoires, show limited long-term stability for complex anaerobic microbial communities, and face substantial challenges in experimental standardization and high-throughput scaling. Furthermore, while strategies such as “engineered consortia” and “postbiotics” currently remain largely preclinical or emerging concepts, integrating these technological innovations with microphysiological systems helps compensate for the systemic limitations of traditional mouse models. They represent a promising framework for driving the future development of precision immunonutrition and personalized microbiome therapy, potentially offering more controllable and safer translational pathways [63].

A critical barrier to translating these findings is the high level of inter-individual and population-level variability in the human microbiome. Th17-associated taxa and metabolite signatures are highly divergent across geographically and ethnically distinct cohorts. Additionally, current clinical datasets are heavily constrained by confounding variables (such as diet and medications) and methodological differences—such as the reliance on low-resolution 16S rRNA sequencing versus high-resolution shotgun metagenomics. These technological and biological variations limit the generalizability of specific microbial biomarkers and underscore the need for standardized analytical pipelines.

The left panel illustrates the systemic decompensation triggered by “evolutionary mismatches.” Modern environmental stressors, specifically Westernized diets and antibiotic overuse, act as disruptive triggers (lightning bolts) that deplete the ancestral butyrate-producing bacteria. This metabolic exhaustion (indicated by low battery icons) compromises epithelial energy supply and mucus integrity, resulting in a “leaky gut” state. Subsequent systemic translocation of lipopolysaccharide (LPS) and pro-inflammatory mediators facilitates the penetration of the blood–brain barrier (BBB). In genetically susceptible hosts, this neuro-immune crosstalk can drive autoimmune pathologies, such as multiple sclerosis (MS), which is often exacerbated by molecular mimicry between microbial antigens and neural proteins. The right panel depicts next-generation microecological strategies designed to repair ruptured symbiotic pacts. Ecosystem-level restoration is achieved through fecal microbiota transplantation (FMT) using “super-donor” profiles, complemented by precision immunonutrition to replenish metabolic niches. At the molecular level, engineered microbial consortia serve as in situ factories for the targeted release of short-chain fatty acids (SCFAs) and other anti-inflammatory effector molecules. Furthermore, postbiotics (including exosomes and purified metabolites) offer a cell-free, pharmacokinetically controllable pathway for the direct delivery of safe immunomodulatory signals to the host mucosal and systemic interfaces. Visual Logic: Red arrows and icons denote the pathobiont-driven inflammatory cascade; green elements symbolize the restoration of metabolic homeostasis and immune tolerance.

## 5. Comprehensive Analysis: The Evolutionary Synthesis and Translational Logic

### 5.1. Macro-Evolutionary Comparison: Scaling of Recognition and Dependence

Table 2 provides a high-level comparison of the three evolutionary nodes instead of simply repeating previous sections. This framework illustrates the “co-evolutionary outsourcing” of developmental cues as complexity increases. Note: The categories *represent interpretive comparative summaries of distinct model systems rather than sequential evolutionary stages.*

### 5.2. The Pathology of Evolutionary Mismatch: A Multi-System Decompensation

**Figure 4:**
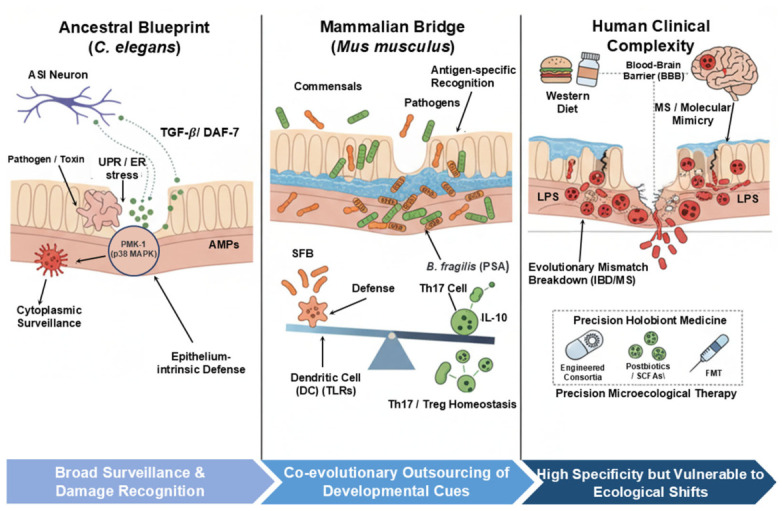
Evolutionary Trajectory of Holobiont Defense: From Ancestral Surveillance to Precision Medicine. Figure legend: Solid black arrows. Abbreviations: AMPs, antimicrobial peptides; ASI, amphid single-ciliated neuron; BBB, blood–brain barrier; DAF, Dauer formation-related; DC, dendritic cell; ER, endoplasmic reticulum; FMT, fecal microbiota transplantation; IBD, inflammatory bowel disease; IL, interleukin; LPS, lipopolysaccharide; MAPK, mitogen-activated protein kinase; MS, multiple sclerosis; PMK-1, p38 MAPK family; PSA, polysaccharide A; SCFAs, short-chain fatty acids; SFB, segmented filamentous bacteria; TGF-β, transforming growth factor-beta; Th17, T helper 17; TLRs, Toll-like receptors; Treg, regulatory T; UPR, unfolded protein response; MHC, major histocompatibility complex; PRR, pattern recognition receptor. Figures were created with Figdraw. The figure synthesizes the evolutionary trajectory of host–microbe interactions across three pivotal stages. (Left) The ancestral blueprint in *C. elegans* exemplifies “surveillance immunity,” in which pathogens or toxins are sensed indirectly via intracellular stress (UPR/ER stress), triggering the p38 MAPK (PMK-1) signaling hub to induce antimicrobial peptides (AMPs). This local epithelial defense is integrated into a systemic response through the ASI neuron-derived DAF-7/TGF-β neuro-endocrine axis. (Center) The mammalian bridge represents the integration of innate (TLRs) and adaptive immunity. Specific commensals (e.g., SFB and *B. fragilis*) act as developmental instructors, calibrating the homeostatic seesaw between RORγt+ Th17 cells and Foxp3+ Treg cells to balance defense with self-tolerance. (Right) Human Clinical Complexity illustrates the modern “evolutionary mismatch.” Environmental stressors like the Western diet disrupt this ancient pact, leading to barrier breakdown, systemic LPS translocation, and distal pathologies such as Multiple Sclerosis (MS) mediated by molecular mimicry and BBB penetration. Precision Medicine Hub: The inset depicts the toolkit for holobiont restoration, where Engineered Consortia, Postbiotics (SCFAs), and FMT serve to re-establish the microbial “navigation” lost in modern environments. Visual logic: Solid black arrows trace intracellular signaling flow; green dotted lines denote remote neuro-immune integration; and the horizontal chevrons (bottom) summarize the shift from broad-spectrum damage recognition to high-specificity, microbiota-dependent immune networks.

The friction between million-year-old immune programs and the rapid shifts in modern industrialization defines the “evolutionary mismatch” that is currently destabilizing human health [67]. As conceptualized in Figure 4, the pathology is not merely a single failure of defense, but a systemic decompensation triggered by the degradation of ancestral microbial “navigation.”

Central to this collapse is the erosion of the intestinal “firewall.” Modern ecological stressors—specifically, Westernized diets and indiscriminate antibiotic exposure [68]—alter microbial diversity and actively impair homeostatic efferocytosis, allowing the systemic translocation of immunogenic lipopolysaccharide (LPS) into the host’s internal environment [69]. This breach at the mucosal interface deprives the immune system of the essential “educational” signals required for developmental calibration [70]. Without this symbiotic frame of reference, the immune network often fails to establish a proper rheostat, leading to an increase in pediatric allergies as conceptualized by the hygiene hypothesis (or the “old friends” hypothesis) and the characteristic Th17/Treg polarization shifts observed in chronic inflammatory states [71].

Crucially, the repercussions of this mismatch extend beyond the gut. The resulting “leaky” immune state resonates across distal physiological axes, shaping host outcomes ranging from central nervous system (CNS) resilience and metabolic stability [72] to the therapeutic efficacy of modern cancer immunotherapies [73]. Viewed through this lens, chronic non-communicable diseases are symptomatic of a holobiont struggling to adapt to a habitat that no longer provides the necessary co-evolutionary cues.

### 5.3. Translational Frontiers: Precision Tools for Holobiont Restoration

To correct the deviations caused by modern lifestyles, precision microbiome therapy must move beyond broad interventions toward molecular-targeted repair. Table 3 summarizes the maturity and evidence strength of these strategies.

### 5.4. Limitations of Current Frameworks and Model Systems

This review’s comparative approach has several limitations. First, translating immunological findings from *C. elegans* and inbred mouse models to the highly heterogeneous human population remains challenging. Second, much of the human clinical data on microbiome-immune associations remains correlative rather than causative. Third, the inter-individual variability of the human microbiome and confounding factors (such as diet and drug history) limit the generalizability of some conclusions. Finally, the holobiont framework is a simplified conceptual tool, and its evolutionary validity regarding group selection remains debated.

## 6. Concluding Remarks: Toward Holobiont Medicine

The evolution of the immune system reflects a fundamental transition from a localized physical barrier to a sophisticated cross-kingdom communication interface. By tracing the trajectory from the primitive surveillance immunity of *C. elegans* to the intricate mammalian adaptive networks, it becomes evident that immune homeostasis is not an autonomous host process but a collaborative achievement of the holobiont. Deconstructing this interaction blueprint allows us to re-examine modern inflammatory diseases as a systemic decompensation triggered by the “evolutionary mismatch” between our ancestral genomes and industrialized environments. Moving forward, the clinical paradigm must shift from a host-centric model of “pathogen eradication” toward one of “symbiotic modulation.”

By integrating precision interventions, such as engineered consortia, postbiotics, and personalized immunonutrition, clinical practice can move beyond mere immunosuppression to actively support mucosal barrier integrity and immunological tolerance. To successfully translate these principles into clinical practice, the future research agenda must focus on: (1) establishing robust causal validation of specific microbe-immune interactions using gnotobiotic and humanized models; (2) integrating multi-omics datasets with patient-derived organoid systems to bypass species translation barriers; (3) conducting stratified clinical trials that account for inter-individual microbiome variability; and (4) standardizing the formulation of next-generation interventions to ensure safety and reproducible efficacy. This integrated approach, which considers the host and its associated microbiota as a functionally coordinated system, provides a promising foundation for addressing chronic inflammatory challenges in the 21st century.

## Figures and Tables

**Table 1:** Summary of key human clinical cohorts and therapeutic interventions for immune-mediated and infectious disorders.

Disease/Indication	Study Type/Cohort	Sample Size	Key Intervention/Association	Main Outcome	Ref
**Ulcerative Colitis** (**IBD**)	Randomized Controlled Trial (RCT)	N=103	Multi-donor FMT vs. Placebo	Significant clinical remission; associated with increased short-chain fatty acid (SCFA) producers.	[52]
**Multiple Sclerosis** (**MS**)	Cohort Study	N=60 (twins)	Microbiota profiling	MS-derived microbiota transfers to mice exacerbate EAE; identified specific enrichment of *Akkermansia*.	[58]
**Clostridioides difficile** (**CDI**)	Clinical Trial	N=116	Lyophilized oral FMT	High rate of resolution for recurrent CDI compared to standard antibiotic therapy.	[53]

**Table 2:** Evolutionary Scaling of Immune Recognition and Microbial Dependence.

Feature	Ancestral (Worms)	Mammalian Bridge (Mice)	Human Clinical Complexity
**Recognition Mode**	Cytoplasmic Surveillance (monitoring proteostasis/mitochondria) [64]	Surface PRR/MHC (diversified receptor diversification) [65]	Epigenetic/Metabolic Calibration (long-term memory hubs) [66]
**Microbial Role**	Metabolic signal partners (e.g., NO)	Essential developmental instructors (e.g., SFB/Th17)	Threshold-setting “symbiotic frame of reference”
**Vulnerability**	High metabolic cost/Non-specific damage	Sensitivity to specific dysbiosis	Evolutionary Mismatch [67]

**Table 3:** Precision Intervention Toolkit and Evidence Maturity.

Intervention	Biological Logic	Representative Targets/Evidence	Maturity Level
**FMT**	Ecosystem Restoration	Super-donor selection; Phage-bacteriome interactions	High (for recurrent CDI); Moderate/Mixed (for IBD)
**Engineered Consortia**	Targeted Effector Release	In situ delivery of anti-inflammatory molecules [73,74]	Pre-clinical/Emerging
**Postbiotics**	Molecular Signaling	SCFAs, urolithin A, and membrane vesicles [74,75]	Early Clinical/Pre-clinical
**Precision Nutrition**	Epigenetic Calibration	Macronutrient/Polysaccharide substrate targeting [76,77]	Emerging [78]

## Data Availability

No new data were created or analyzed in this study. This is a review article, and no new experimental data were generated. All data cited in this article have been referenced to their original sources, and readers may refer to the corresponding references for the original data.
